# Implementing ICHOM standard set for cataract surgery at IPO-Porto (Portugal): clinical outcomes, quality of life and costs

**DOI:** 10.1186/s12886-021-01887-6

**Published:** 2021-03-05

**Authors:** Lara Queirós, Patrícia Redondo, M. França, Sérgio Estrela Silva, Pedro Borges, António Benevides de Melo, Nuno Pereira, Paulo Freitas da Costa, Nazaré Carvalho, Marina Borges, Isabel Sequeira, Francisco Nuno Rocha Gonçalves, José Lemos

**Affiliations:** 1 Department of Ophthalmology, IPO-Porto, Porto, Portugal; 2 Management, Outcomes Research, and Economics in Healthcare Group, Porto, Portugal; 3 Outcomes Research Lab, Instituto Português de Oncologia do Porto Francisco Gentil (IPO-PORTO), Rua Dr. António Bernardino de Almeida, 4200-072 Porto, Portugal; 4 Quality and Patient Safety, IPO-Porto, Porto, Portugal; 5 Luz Saúde, Lisbon, Portugal; 6 MEDCIDS/FMUP, Hospital de São João 9623, 4200-450 Porto, Portugal

**Keywords:** Outcomes assessment, Ophthalmology, Cataract, Patient-reported outcomes measures

## Abstract

**Background:**

This paper fills a gap in the applied research field, for a local context, by addressing the topics of describing cataract surgery’ clinical outcomes; quality of life (QoL); and costs of the patients treated after the implementation of the ICHOM standard set.

**Methods:**

This is a retrospective observational study using real-world data (RWD). We included all patients subjected to cataract surgery at the Portuguese Institute of oncology - Porto (IPO-Porto), Portugal, after 3 months follow up period completed between 5th June 2017 and 21st May 2018. The following inclusion criteria: corrected visual acuity of ≤ 6/10 or other significant visual disturbance due to lens opacity or the existence of a large anisometropia. A circuit was implemented based on the ICHOM standard for cataract, to measure clinical variables (e.g. visual acuity) and QoL (CATQUEST-9SF) before and after surgery, and cost of treatment. The results were explored by means of a paired-sample t-test, considering normality assumptions.

**Results:**

Data refers to 268 patients (73 P25-P75:32–95 years old), regarding 374 eyes. The cataract surgery had a positive effect on visual acuity (*p* < 0.001), refraction (right and left cylinder; *p <* 0.001) and all QoL dimensions. The vast majority of patients, around 98%, reported improvements in QoL. Based on IPO-Porto administrative records, the direct cost of treating cataracts (per eye) is of 500€, representing a total cost of 187,000€ for the number of patients operated herein.

**Conclusion:**

This study reports the successful implementation of the ICHOM standard set for cataracts in a Portuguese institution and confirms that cataract surgery provides a rapid visual recovery, with excellent visual outcomes and minimal complications in most patients, while also having a positive impact on patients’ quality of life.

## Background

Cataract is an opacity of the lens that reduces the amount of incoming light and results in visual function deterioration. Age is the predominant risk factor for cataract formation. Other relevant risk factors in our institution include the history of radiotherapy, chemotherapy and corticosteroid drug use [1].

Currently, cataract is considered the most prevalent cause of blindness worldwide [2], with an adverse impact on patient’s quality of life [3]. The World Health Organization (WHO) estimated that there were 95 million people visually impaired due to cataracts in 2014 and that the number of cataract blind people will reach 40 million in 2025 [4]. The most recent Global Burden of Disease (GBD) study showed that cataracts (8.0 million DALY) had the second burden among eye diseases, pathologies with cumulative 29.9 million DALYs in 2017 [5]. Cataract burden is on the same range of measles, gastritis/duodenitis and cervical, prostate and pancreatic cancers [5]. Its presence is associated with impaired work ability [6] and increased mortality, which might be due to the link with age and systemic conditions such as type 2 diabetes mellitus or smoking [7].

Nevertheless, the prevalence of cataracts has been declining for the past two decades due to increasing rates of cataract surgery with improved techniques and active surgical initiatives [8]. Cataracts are not yet preventable, and surgery remains the only effective treatment with a high success rate in improving visual function with low morbidity and mortality [9].

Cataract surgery is one of the most cost-effective treatments, and the most commonly used procedure in many countries [10, 11]. In the European Union (EU), Portugal, is the country with the highest rate of cataract surgeries, with 14 surgeries per one thousand inhabitants [12]. Moreover, the number of cataract surgeries increased from 14,226 in 1993 to 146,958 in 2015 [12].

The socioeconomic impact of cataract surgery is significant. It is estimated that it allows people to increase their economic productivity by up to 1500% of the cost of surgery during the first postoperative year [8]. Additionally, several studies have shown that cataract surgery is associated with improvement of visual function and psycho social health status [13, 14], reduction of falls [15, 16], lower prevalence of hip fractures [17], and improvement in quality of life (e.g. social and emotional aspects) [13, 18], among others.

Despite the high effectivity of cataract surgery, treatment rates and outcome assessment vary substantially between ophthalmologic institutions in different countries, limiting direct comparisons and studies about best eye care practices [19]. Recent literature has been focused on finding the best strategies to achieve better outcomes for the lowest cost, maximizing value for patients - value-based healthcare (VBHC). In VBHC, the “value” is derived from measuring health outcomes, especially those that matter to patients, against the cost of delivering them. Assessing these outcomes is a means to compare performance between institutions and can be used to improve healthcare delivery [20]. Recently, outcome reporting turned mandatory in many healthcare institutions and has been incorporated into good medical practices [21]. Nevertheless, many institutions have not presented organized data and determining which clinical outcomes achieve better value for the patient is still challenging [22, 23].

To answer the need for standardized and internationally accepted outcome measures, the International Consortium for Health Outcomes Measurement (ICHOM) [24] developed a set of recommendations for several diseases, including cataracts. The standard sets are developed using a consensus-based process involving extensive consultation with experts (e.g. clinicians, measurement researchers, and patient representatives) and incorporating existing patient-reported outcome measures (PROMs) instruments and new measurement items. They are elaborated to cover the full patient care cycle (including non-surgical and surgical treatment), can be applied in different healthcare settings and recommend a minimum time point for patient data collection [25]. The ICHOM standard set for cataracts was released in 2015, establishing a set of parameters to evaluate the patient with a diagnosis of cataracts, including a presurgical assessment, surgical data, and postoperative outcomes [25].

To our knowledge, these standards are not fully implemented in any Portuguese public institution. IPO-Porto is an oncology hospital, which carries out approximately 900 cataract surgeries/year, mainly in patients with senile cataracts and cataracts due to oncology treatments. The specificity and complexity of these patients increase the need to measure disease outcomes adequately. However, until 2017, the IPO-Porto Ophthalmology Service did not have these data organized, and adequate outcome measurement was a challenge.

The purposes of the present study were to describe cataracts-related clinical outcomes, quality of life and costs of the first patients with cataracts who were treated after the implementation of the ICHOM standard set.

## Material and methods

This is a retrospective observational study using real-world data (RWD) from patients who have been submitted to cataract surgery at IPO-Porto after implementation of the ICHOM standard set.

The study protocol was approved by the IPO-Porto Ethics Committee (CES.381/018).

### Development and implementation of the ICHOM circuit at IPO-Porto

**Fig. 1:**
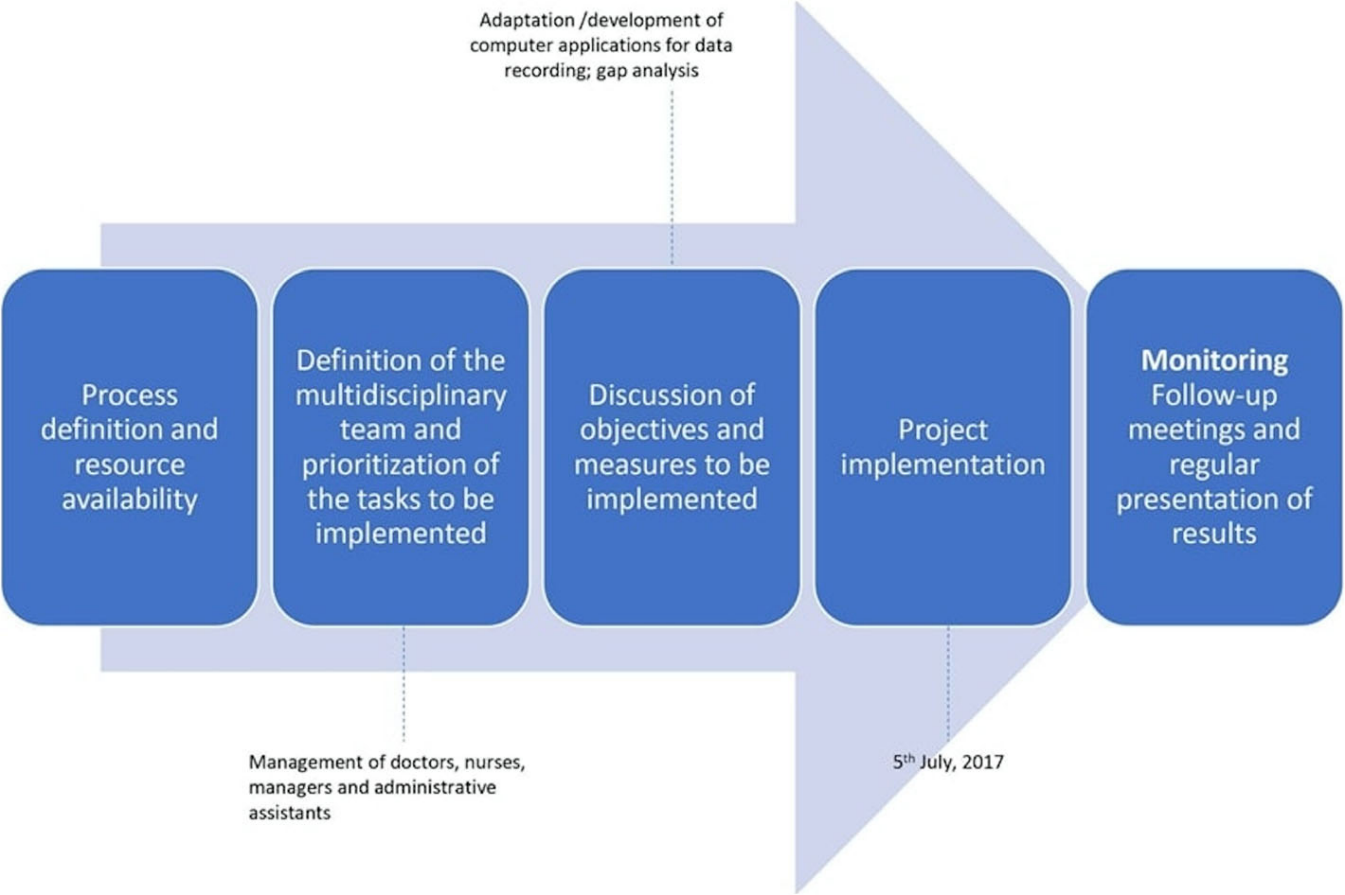
Methodology defined to implement ICHOM Standard Set

In 2017, IPO-Porto designed a pathway to implement the ICHOM standard set for cataracts into the daily practice (Fig. 1). The development and implementation of the IPO-Porto ICHOM circuit were performed following the steps below:

#### Gap analysis

A gap analysis was performed, aiming to provide the project team with an understanding of the differences between current practices and the best practice (ICHOM Standard Set), in order to understand to what extent cataract diagnosis and monitoring in the current service differs from the ICHOM Standard Set.

#### Definition of the working group

**Fig. 2:**
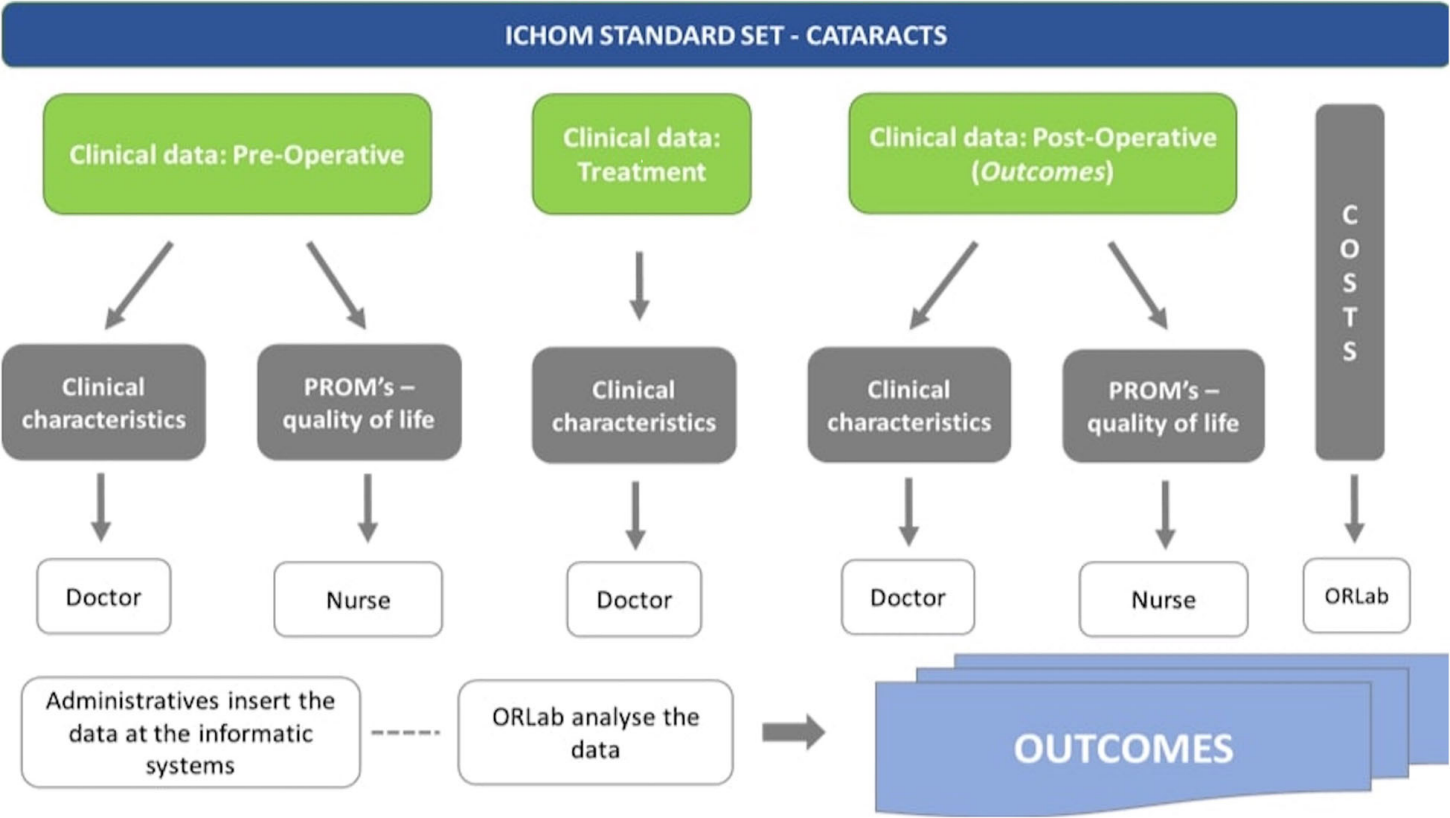
Circuit implemented by the IPO-Porto

In order to engage the organization, teamwork was convened, which involved three members of the board of directors: the director and nurse responsible for the service and a member of the Outcomes Research Lab (ORLab), in order to outline the best strategy for the implementation of a data collection circuit. Once the circuit was defined (Fig. 2), the ORLab developed the support material to be assigned to the Ophthalmology Service, to backup the data collection related in the project, and to monitor/follow-up the entire circuit.

##### ICHOM standard set for cataracts

The ICHOM standard set for cataracts is a free standard set that can be used for free by any institution. It establishes a set of standardized parameters to evaluate cataract patient ocular health before and 3 months after treatment (cataract surgery). The ICHOM standard set focus on four major clinical points: patient-reported visual functioning (e.g., tracked via the CATQUEST-9SF), major surgical complications (e.g., capsular problems, dropped nucleus or lens fragments into the vitreous, return to the operating theater, endophthalmitis, persistent corneal edema, among others), refractive error and visual acuity. Furthermore, the ICHOM standard also supports the relevance of measuring the costs of treatment as a crucial step to evaluate the gap between demand and capacity of healthcare service. In ICHOM costs are regarded as the actual use of resources involved in a patient’s care process and are computed based on the following data: the time devoted to each patient by the care resources; the capacity cost of each resource; and the support costs required for each patient-facing resource [24].

The standard set for cataracts contains a comprehensive set of variables, available at the ICHOM website: https://www.ichom.org/portfolio/cataracts/. All these clinical and cost-related aspects and variables were included in the IPO-Porto ICHOM circuit.

##### Patient selection

All patients submitted to cataract surgery at IPO-Porto, with 3 months follow up period completed between 5th June 2017, and 21st May 2018 were included. The following inclusion criteria were considered: corrected visual acuity of ≤ 6/10; the existence of a large anisometropia or the existence of a manifest lack of visual quality due to lens opacity. Pediatric cataracts were excluded.

##### Data collection

**Fig. 3:**
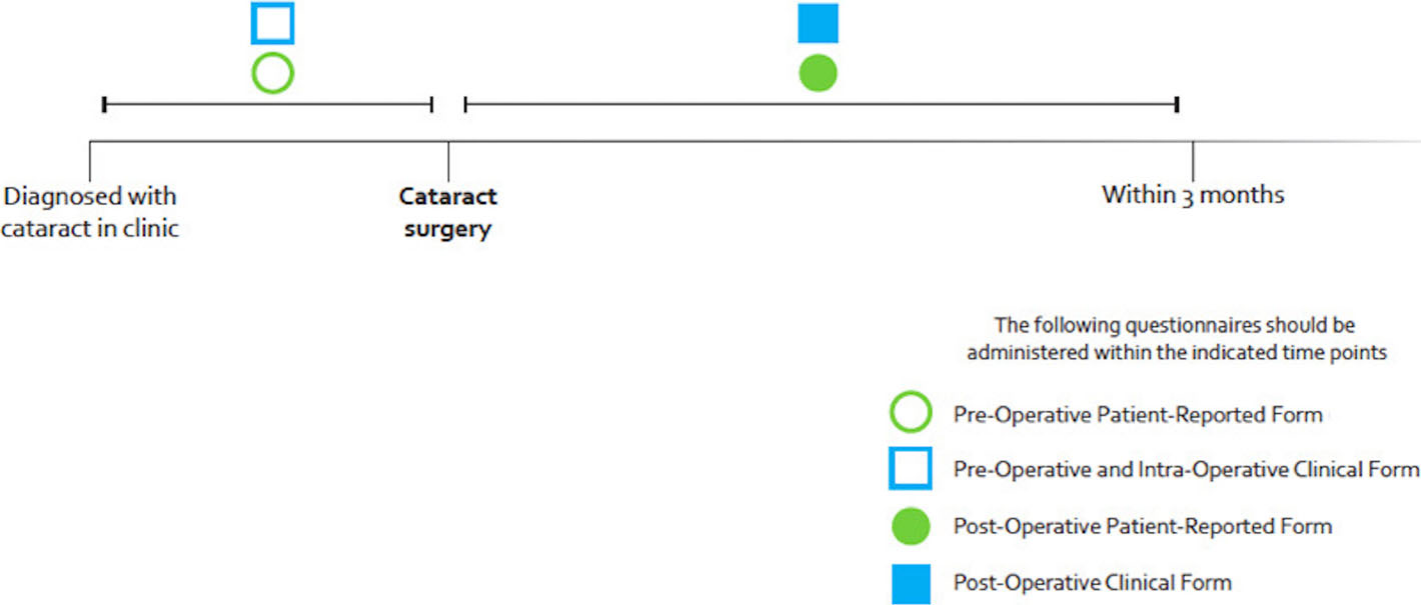
Follow-up timeline by the ICHOM [24]

Data collection was based on the timeline established by ICHOM and is represented in Fig. 3 [24].

Physicians were responsible for collecting clinical data (pre/intra/postoperative) while nurses were responsible for applying the CATQUEST-9SF questionnaire before - and 3 months after cataract surgery.

CATQUEST-9SF is a patient-reported outcome questionnaire, which comprises nine items to measure activity limitation in patients’ daily life because of vision before and after their cataract surgery. It comprises 2 global assessment questions about the patients’ difficulties in general and their satisfaction with vision and 7 questions of a perceived difficulty in performing daily-life activities. Each of these items has four response options: 4 = ‘Yes, very great difficulties’; 3= ‘Yes, great difficulties’; 2= ‘Yes, some difficulties’; and 1 = ‘No, no difficulties’. For the global question about the patients’ satisfaction with their vision, the response categories are as follows: 4 = ‘Very dissatisfied’; 3 = ‘Rather dissatisfied’; 2 = ‘Fairly satisfied’; and 1 = ‘Very satisfied’. All items contain an additional option ‘Cannot decide’. Scoring is computed using a Rasch score [26]; Rasch modelling provides a method to transform ordinal data (e.g. data from Likert-type items) into continuous, equal interval units (logits), which allows for the summation of the items’ raw scores, where the summed raw score is a sufficient statistic [27]. It takes into account that the items are of varying difficulty and that the distance between the response options is not equal. The option “Cannot decide” is considered as missing in the Rasch analysis.

The medical records and CATQUEST-9SF results from the IPO-Porto Ophthalmology Service were written on paper and maintained in folders. After the appointment, administrative assistants were responsible for registering the collected data on a structured electronic database.

##### Cataract surgery costs

Data on costs of cataract treatment were collected by the ORLab using IPO-Porto administrative records. The average cost of each cataract surgery (performed by two surgeons using phacoemulsification) was calculated considering a healthcare provider perspective and accounting for the following procedures:

### Preoperative

- One nurse appointment;
- One doctor appointment;

### Intraoperative

- Surgery (including clinical consumption materials and human resources - two surgeons, two nurses and one auxiliary nurse);
- One nurse appointment;
- One doctor appointment;

### Postoperative

- One nurse appointment;
- One doctor appointment;

We accounted for: human resource costs, considering the value/hour for each professional class; costs with consumables and drugs used in surgery; and if surgery was scheduled or additional. Costs related to surgery complications and indirect costs were not considered in this analysis. The cost data is presented in euros (€).

### Statistical analysis

Categorical variables were described as relative frequencies (%) and mean, and standard deviation (sd) were used for continuous variables. To compare refraction target, visual acuity and Catquest results pre- and post-surgery, a paired-sample t-test was applied, taking into account normality assumptions. The significance level was set at 5% (*p* < 0.05) for all tests. All analyses were performed using the SPSS® software (*Statistical Package for the Social Sciences*), v.25.0.

## Results

This study included 268 patients, consisting of 374 operated eyes, 235 eyes (62.8%) in women. The median age was 73 (P25-P75:32–95) years old (Table 1).

**Table 1:** Preoperative demographic patient characteristics

	N° of cases (eyes operated) (***n =*** 374)
**Age (in years), median (P25-P75)**	73 (32–95)
**Sex, female**	235 (62.8%)
**male**	139 (37.2%)

Table 2 presents the clinical characteristics of the included patients. A total of 187 right eyes were operated during the study period.

**Table 2:** Preoperative clinical characteristics. Results are presented as percentages (%) of operated eyes (*n =* 374), except when otherwise indicated

	%
**Operated eye (left),** ***n***	187
**Ocular comorbidities in the operated eye**	
Glaucoma	3.5
Macular degeneration	3.5
Diabetic retinopathy and/or diabetic macular edema	1.1
Amblyopia	1.1
Other	6.4
**Prior ophthalmic interventions**	
Cataract surgery on the fellow eye	48.7
Corneal refractive surgery on the operative eye	1.3
Vitrectomy on operative eye	0.5
Other intervention on operative eye likely to negatively impact the clinical outcome	0.8

A total of 4.5% of patients had a white and dense brown cataract in the operated eye. Intraprocedural complications occurred in less than 1% of the patients; the most frequent being zonular dehiscence and vitreous prolapse, each in 0.8% of the patients (Table 3).

**Table 3:** Intraoperative clinical patient characteristics (*n =* 268). Results are presented as percentages (%), except when otherwise indicated

	%
**Surgery (Phacoemulsification),** ***n***	100
**Technical factor on operated eye**^**a**^	
White or dense brown cataract	4.5
Pseudoexfoliation	3.5
Corneal opacities (severe)	1.1
Pupillary problems (severe)	2.1
**Intrasurgical Complications**	
Capsule breach	0.5
Zonular dehiscence	0.8
Vitreous prolapse	0.8
Lens fragments into vitreous	0.0
Other	0.0

^a^Intrinsic factors / Characteristics of the ocular anatomy or physiology that have the potential to increase surgical challenge and lead to higher chance of complications

The comparison between refraction and visual acuity in pre- and post-surgery patients are presented in Table 4. Visual acuity has significantly increased post-surgery. Refraction (target/actual) was significantly increased in both the right and left cylinder post-surgery, but no statistically significant difference was noted for spherical right and left refraction.

**Table 4:** Pre- and post-surgery refraction and visual acuity

Visual acuity	Pre-op (mean)	Post-op (mean)	Mean Dif.	***p-***value^**1**^
Best-corrected right	0.4240	0.8868	−0.4627	< 0.001
Best-corrected left	0.4325	0.8682	−0.4357	< 0.001
**Refraction (target/actual)**	**Pre-op** **(mean)**	**Post-op** **(mean)**	**Mean Dif.**	***p-*value**^**1**^
Spherical right	−0.3789	−0.3161	− 0.0629	0.293
Spherical left	−0.4104	−0.4156	0.0152	0.850
Cylinder right	−0.3115	−1.0496	0.7382	< 0.001
Cylinder left	−0.4123	−0.9844	0.5721	< 0.001

1- Paired-sample T-test

Table 5 presents the observed postoperative complications. Less than 1% of the operated patients had to return to the operating room within 3 months of the surgery. The most common post-surgery complication was corneal edema (0.5%); 0.3% of patients had endophthalmitis.

**Table 5:** Postoperative complications. Results are presented as percentages (%)

	%
**Postoperative Complications**	
Return to operation theater within 3 months	0.8
Endophthalmitis	0.3
Corneal edema	0.5
Other	1.3

**Fig. 4:**
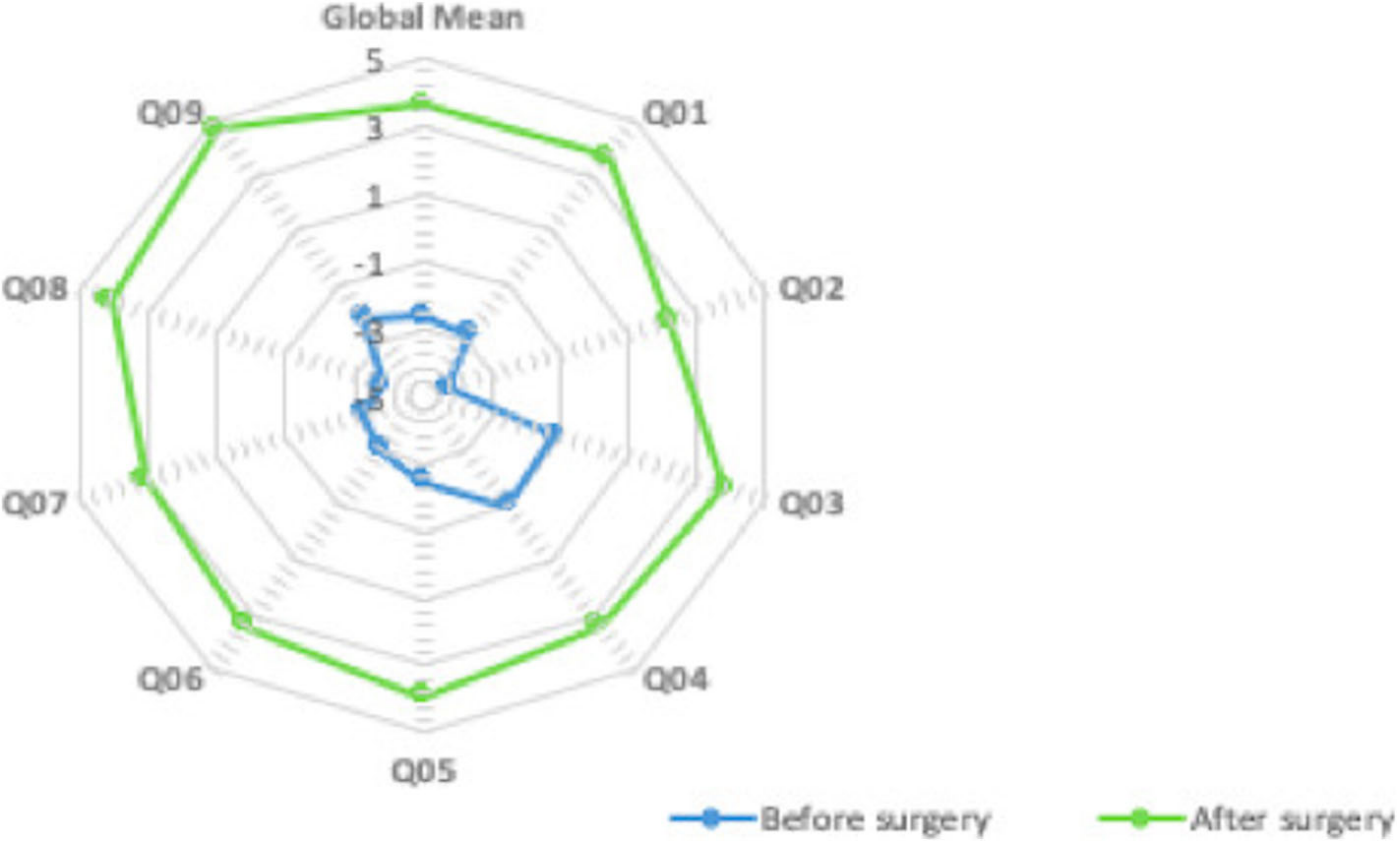
Radar chart representing the quality of life, assessed with CATQUEST-9SF, before and after surgery (*n* = 374). Dimensions of the questionnaire: Q01 = Sight at present causes difficulty in everyday life; Q02 = Satisfaction related to sight at present; Q03 = Difficult level reading text in newspapers; Q04 = Difficult level recognizing the faces of people meet; Q05 = Difficult level seeing the prices of goods when shopping; Q06 = Difficult level seeing to walk on uneven surfaces, e.g. cobblestones; Q07 = Difficult level seeing to do handicrafts, woodwork, etc.; Q08 = Difficult level reading subtitles on TV; Q09 = Difficult level seeing to engage in an activity/hobby that you are interested in

Cataract surgery had a positive effect on all quality of life dimensions (Fig. 4). Around 98% of patients reported a significant improvement in the quality of life (*p* = 0.000).

Table 6 presents the procedures performed in patients with cataracts and their respective costs. The direct cost of treating one cataract is approximately 500€, representing a total cost of 187,000€ for the number of included patients.

**Table 6:** Cataract-related procedures performed in these patients and their costs

Preoperative	Total cost per eye (€)
Nurse appointment	6.47
Doctor appointment	28.98
**Intraoperative**	
Doctor appointment	28.98
Nurse appointment	6.47
Clinical material and drugs	236.86
Surgeons (*n =* 2)	54.45
Surgical nurses (*n =* 2)	10.79
Auxiliary nurse (*n* = 1)	2.52
**Postoperative**	
Nurse appointment	6.47
Doctor appointment	28.98
**Total**	**499.09**

## Discussion

This study reports the successful implementation of the ICHOM standard set for cataracts in an ophthalmology unit within an oncology hospital, describing the clinical outcomes, quality of life and cost of treating the first patients. After surgery, we found a significant improvement in visual acuity, refraction and quality of life (in all CATQUEST-9SF dimensions), with a low proportion of intraoperative or postoperative complications. These results corroborate that also in an oncology population, cataract surgery is highly effective, leading to improved vision and quality of life [14, 28]. However, until ICHOM standard set to release, there was no validated tool available, to characterize a specific population such as assessed at IPO-Porto.

WHO estimated that there were 95 million people visually impaired due to cataracts in 2014 [29]. Previous studies have reported that the prevalence of cataract increases with age [8, 30, 31]. In our study, including population from a particular setting - an oncology hospital -, with a higher risk for cataracts secondary to cancer treatments, the median age was 73 years, similar to other studies [32–34].

Since its foundation, ICHOM has developed comprehensive standards sets for several diseases, encouraging broader measurements of outcomes and collaboration concerning global outcome comparisons [35]. The standard sets should be applied in both routine clinical practice and clinical studies in order to standardize the measured outcomes worldwide [36], enabling global comparisons and benchmarking and driving improvements in relevant patient outcomes [35, 37]. Currently, several healthcare institutions have adopted ICHOM standards for different conditions, such as pregnancy and childbirth [38], breast cancer [39], hip and knee osteoarthritis [25]. Although efforts in this regard have increased, there is still a limited understanding of how these standard sets perform in clinical settings, as few implementations are described in the literature.

The implementation of the ICHOM standard set for cataracts at the IPO-Porto provides insight into the results of cataract surgery within the institution. It allows global comparisons, improving knowledge on the unmet needs of cataracts management and being a basis for the provision of better patient care. The implementation of this standard set for the evaluation of patient outcomes is not only innovative within the institution, but also in line with a new wave of innovative learning in this area in Portugal and other developed countries, profiting from the value created by health services.

Therefore, this study provides relevant information on the practical implementation of an ICHOM standard set. Based on this experience, we observed that this implementation process requires a highly motivated team, with close communication between the elements and a pragmatic approach that allows the integration of these procedures with daily routines, which is essential for its success. Also, the implementation of the cataract standard set allows for a holistic evaluation of the value created within the Ophthalmology Service, with increased patient engagement.

Although institutions understand that shared metrics are the first step to improve the quality of data, international standard implementation is still a challenge in many contexts. A recent study reported the inconsistency of ophthalmological outcome measures reported in eight eye hospitals worldwide. Although several hospitals have reported similar outcomes, little congruence was verified concerning which outcomes should be reported, which methodologies should be used and how to address preoperative risks and co-morbidities [19].

The ICHOM standard set allowed a better assessment of the results of cataract surgery patients from IPO-Porto in a simple way and provides a good basis for comparisons with other institutions in Portugal and globally. In addition, using the CATQUEST-9SF questionnaire, we demonstrated that, cataract surgery is associated with high self-reported patient satisfaction, with significant improvements in their QoL, which is particularly relevant in cancer patients. These results are in line with those reported by Chen et al. demonstrating that postoperative visual function outcomes after cataract surgery achieved the expected level of improvement in the majority of cataract patients [40].

The present study has shown the practical experience and outcomes of an implementation of the ICHOM standard set for cataracts in Portugal. Although we determined that the cataract surgery cost (per eye) was 500€, we understand that this cost varies substantially depending on the country and care setting [35]. For instance, the mean total costs per cataract intervention varies considerably from country to country, ranging from 318€ in Hungary, 1087€ in Italy to $2691.98 (2442.56€) in the US [41, 42]. Nevertheless, direct comparisons with our findings are difficult because of different methodologies in cost estimations. Also, intraocular lens choice has a significant impact on the overall cost. Despite these differences in cost assessment, cataract surgery is ranked as the most cost-effective intervention, with 4500% financial return on investment [43].

Finally, the IPO-Porto team was able to apply the results of this study to improve daily clinical practice. Before ICHOM implementation, patient assessment was not standardized and, during clinical appointments, the health professional only obtained a subjective patient evaluation collected without a validated tool. Following this study, a validated questionnaire (CATQUEST-9SF) was included in the daily clinical practice to systematically and objectively assess disease specific, patient-reported quality of life. This dimension of the surgical outcome is strikingly important for clinical practice and ICHOM implementation has helped the clinical health team to better understand and value the effects of surgery on the quality of life of these patients. This perspective is absolutely fundamental to modern medicine and plays an even more important role in this specific population.

However, the study presents some limitations. Firstly, these results could not be compared with results prior to ICHOM standard implementation, since these data were not previously collected at IPO-Porto. Second, we did not implement the Time-Driven Activity-Based Costing (TDABC) method, recommended by ICHOM to calculate the cost. Our future perspective is to improve cost analyses applying TDABC methodology. Moreover, in future works, with larger samples, we intend to perform stratified analysis to assess possible differences regarding quality of life between groups of patients.

## Conclusions

This study reports the successful implementation of the ICHOM standard set for cataracts in a Portuguese institution, reinforcing the positive impact the intervention has on quality of life and other relevant clinical outcomes.

The inclusion of patient-reported outcomes is a crucial point, enabling people to report directly regarding their disease and the effect of the surgery in their daily life. Implementation of standards in medicine is still a significant challenge as, not infrequently, it increases the workload for the doctors, diverting their time from patient care. EHR with structured data fields which don’t require duplication of clinical records can prevent this.

Even without this optimized implementation, the ICHOM data set was successfully included in the doctors’ clinical routine, without compromising their attention to the patient. Nursing and administrative staff were key elements regarding this point.

The ICHOM data sets implementation leverages the clinically meaningful data collection and sharing among peers in a standardized and consistent way. This supports informed decisions by patients and health care providers and delivers long-term benefits to the population contributing to eye care improvement globally. This standard set implementation has consolidated acceptable clinical practices without overloading the IPO-Porto service. The cataract standard set is mostly used as a “pilot experiment”, precisely because of its easy implementation and brief follow-up time, it’s the clinical pathway’s consistency, and its high-quality control of the obtained data (few variables and objective questions). For this reason, this standard set is confirmed as an excellent example of knowledge development, and learning from this experience extends this evaluation to the oncological area. In other words, the adoption of this methodology does not announce the end, but the beginning of a new wave of learning that reflects the measurement of the value generated by the provision of health care.

## Data Availability

The datasets used and/or analysed in the current study are available from the corresponding author on reasonable request.

## Abbreviations

DALY: Disability Adjusted Life Years
EU: European Union
GBD: Global Burden of Disease
ICHOM: International Consortium for Health Outcomes Measurement
ORLab: Outcomes Research Lab
PROMs: Patient-reported outcome measures
Qol: Quality of life
RWD: Real-world data
TDABC: Time-Driven Activity-Based Costing
VBHC: Value-based healthcare
WHO: World Health Organization
